# Therapeutic Notes

**Published:** 1896-05-23

**Authors:** 


### THERAPEUTIC NOTES.

### TREATMENT OF SYPHILIS BY THE INJECTION OF SYPHILITIC ANTITOXIN.

Cotterel[1] has recently reopened the question as to the possibility of treating syphilis in the first stages by the injection of serum taken from persons in the secondary and third stages. He maintains that the primary lesion, enlarged glands and early skin eruption, succumb to this treatment more rapidly than when the ordinary mercurial treatment is persevered with; he recommends the injection of ½—5 cubic centimetres of serum taken from persons in the secondary stage, and he regards military hospitals as most likely to afford fruitful fields of research, owing to the number of such cases in these institutions, and the possibility of organised control. Gilbert and Fournier[2] in the early part of the past year gave it as their opinion, in view of our present indefinite knowledge of the subject, that this line of treatment could be safely adopted in most cases. Other observers, namely, Tommasoti, Mazza, Héricourt. and Triboulet, employed a somewhat modified method, They injected their patients with serum from animals, which presumably were additionally immune by reason of a previous course of inoculation with serum from syphilitic subjects. Favourable results were occasionally met with, but complete failure as often followed. Héricourt and Richet[3] have placed on record a few successful cases; one was that of a young woman who had contracted the disorder about three years previously. At the commencement of the serum treatment she possessed an intractable ulcer of the leg. In the course of six days twelve cubic centimetres were injected, and at the end of that period she was suffering from giddiness, headache, pyrexia, and albuminuria. Consequently the injections were suspended with subsidence of the symptoms. The ulcer then rapidly improved, and in one month was completely cured. Héricourt has also got good results in nervous disorders of syphilitic origin, and makes the suggestion that sero-therapy might prove of considerable benefit in cases of tabes of a specific nature. Another case is recorded by the same author, in which broken down gummata readily responded to this treatment after an ineffectual course of mercuric treatment.

[1] Med. Modern Par., 1895, Oct. 12. [2] Sem. Med., Ap., 1895. [3] Compt. Rendu de la Soc. d. Biol., Jan., 1895.

### A PRELIMINARY PRECAUTION BEFORE INDUCING GENERAL ANÆSTHESIA.

In a paper recently published by Fraenkel he explains that for the last 20 years he has had recourse to an expedient in giving anæsthetics which has afforded most excellent results. Since adopting the method he has not once met with any accident, although the patient may have been the subject of cardiac, pulmonary, or other disease. His plan is to give an injection of the following formula a quarter of an hour before the operation commences: Hydrochlorate of morphia, 15 centigrams; hydrate of chloral, 25 centigrams; sulphate of atropine, 15 milligrams; distilled water, 25 c. centimetres. The quantity injected should be between one and one and a-half c. centimetres according to the constitution of the patient. Very excitable or hysterical women, as well as those who indulge in alcohol, stand full doses. The advantage claimed for the method is that a smaller quantity of the anæsthetic, be it chloroform or ether, is subsequently required. In the majority of cases 25 to 30 grams of chloroform are sufficient, and it is unusual to employ more than 50 grams even in operations of long duration. The stage of excitability is short and feebly marked, vomiting is exceptional, and even after prolonged anæsthesia the pulse and respiration remain satisfactory. Finally, the after effects, such as sickness, malaria, and nausea, are far less noticeable than is usual with the ordinary methods.

